# Evolutionary Changes in Vertebrate Genome Signatures with Special Focus on Coelacanth

**DOI:** 10.1093/dnares/dsu012

**Published:** 2014-05-06

**Authors:** Yuki Iwasaki, Takashi Abe, Norihiro Okada, Kennosuke Wada, Yoshiko Wada, Toshimichi Ikemura

**Affiliations:** 1 Department of Bioscience, Nagahama Institute of Bio-Science and Technology, Nagahama, Shiga 526-0829, Japan; 2 Department of Information Engineering, Faculty of Engineering, Institute of Science and Technology, Niigata University, Niigata-ken 950-2181, Japan; 3 Faculty of Bioscience and Biotechnology, Tokyo Institute of Technology, Yokohama, Kanagawa 226, Japan; 4 Department of Life Sciences, National Cheng Kung University, Tainan 701, Taiwan

**Keywords:** big data, epigenetic, SOM, DNA methylation, CG suppression

## Abstract

With a remarkable increase in genomic sequence data of a wide range of species, novel tools are needed for comprehensive analyses of the big sequence data. Self-organizing map (SOM) is a powerful tool for clustering high-dimensional data on one plane. For oligonucleotide compositions handled as high-dimensional data, we have previously modified the conventional SOM for genome informatics: BLSOM. In the present study, we constructed BLSOMs for oligonucleotide compositions in fragment sequences (e.g. 100 kb) from a wide range of vertebrates, including coelacanth, and found that the sequences were clustered primarily according to species without species information. As one of the nearest living relatives of tetrapod ancestors, coelacanth is believed to provide access to the phenotypic and genomic transitions leading to the emergence of tetrapods. The characteristic oligonucleotide composition found for coelacanth was connected with the lowest dinucleotide CG occurrence (i.e. the highest CG suppression) among fishes, which was rather equivalent to that of tetrapods. This evident CG suppression in coelacanth should reflect molecular evolutionary processes of epigenetic systems including DNA methylation during vertebrate evolution. Sequence of a *de novo* DNA methylase (Dntm3a) of coelacanth was found to be more closely related to that of tetrapods than that of other fishes.

## 1. Introduction

Oligonucleotide composition varies significantly among species even with the same genome G+C% and designated as a ‘genome signature’, which characterizes the genomic sequences of individual species.^1–3^ Kohonen's self-organizing map (SOM) is a powerful tool for clustering high-dimensional complex data on one map.^4,5^ For codon and oligonucleotide compositions handled as high-dimensional data, we have modified the conventional SOM to BLSOM.^6,7^ BLSOMs for oligonucleotide compositions can classify genomic fragments (e.g. 100 kb) from a wide range of species according to species, without species information during the BLSOM calculation.^8,9^ BLSOM can also visualize diagnostic oligonucleotides responsible for the species-specific clustering, supporting an efficient knowledge discovery from a big sequence data.

The present paper studies vertebrate genome signatures and their evolutionary changes with special focus on coelacanth. Coelacanth is one of the oldest known living *Sarcopterygii* and of the nearest living relatives of tetrapods.^10^ Therefore, analysis of this genome is believed to provide information concerning molecular evolutionary processes during the transition from fishes to tetrapods, and the genome of African coelacanth (*Latimeria chalumnae*) has recently been sequenced.^11,12^ Oligonucleotide BLSOM for genome sequences from a wide range of vertebrates, including the coelacanth, shows that CG suppression (i.e. under-representation of CG dinucleotide) is the highest among fishes and equivalent to that of tetrapods. The evident CG suppression in the mammalian genomes has been connected with the methylation at C in CG dinucleotide, which is a well-characterized epigenetic maker. Therefore, phylogenetic tree analyses on DNA methylases of fishes and tetrapods have been conducted. The classification and visualization power of the oligonucleotide BLSOM is very high and can efficiently unveil a wide range of genome characteristics from massive amounts of genomic sequences without prior knowledge, and therefore, this unsupervised data-mining method is a powerful and timely tool useful for genome studies in the era of big sequence data obtained from high-throughput DNA sequencers.

## 2. Materials and methods

### 2.1. BLSOM

SOM is an unsupervised clustering algorithm that non-linearly maps high-dimensional vectorial data onto a two-dimensional array of lattice points.^4,5^ We previously modified the conventional SOM for genome informatics to make the learning process and resulting map independent of the order of data input, on the basis of batch-learning SOM (BLSOM).^6,7^ The initial weight vectors were defined by principal component analysis instead of random values, and BLSOM learning for oligonucleotide composition was conducted as described previously.^8^ The map size was chosen assuming that the average number of data per lattice point was 10. Diagnostic oligonucleotides for species-dependent separation were visualized as described previously.^8^ The BLSOM programme was obtained from takaabe@ie.niigata-u.ac.jp.

### 2.2. Sequences

Coelacanth genome sequences were obtained from http://coelacanth.nig.ac.jp, and other genome sequences were obtained from the UCSC ftp site (http://www.ncbi.nlm.nih.gov/genomes/). When the number of undetermined nucleotides (Ns) in a fragment sequence (e.g. 100 kb) exceeded 20% of the sequence, the sequence was omitted from the analysis. In the case where the number of Ns was <20%, the oligonucleotide frequencies were normalized to the length without Ns and included in the analysis. Amino acid sequences of coelacanth Dnmt3a, Dnmt3b and Dnmt1 (GENE ID: R03136, A01856 and A13991) were obtained from the Coelacanth Genome Project (http://coelacanth.nig.ac.jp/).

## 3. Results

### 3.1. BLSOM for six fish genomes

**Figure 1.**
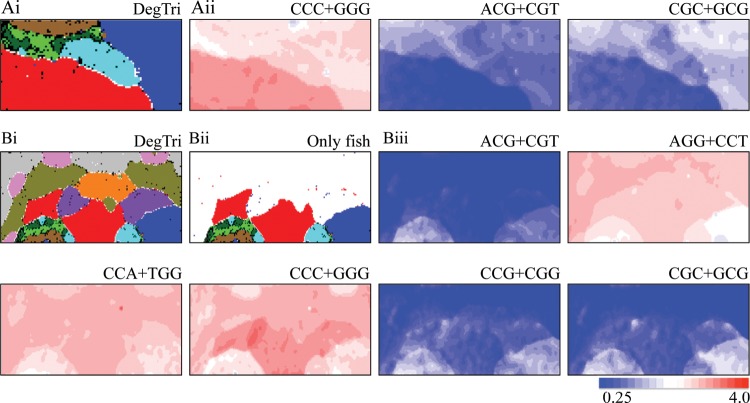
Oligonucleotide BLSOM for 100-kb sequences. (Ai) DegTri for six fish genomes. Lattice points containing sequences from multiple species are indicated in black and those containing sequences from a single species are coloured as follows: coelacanth (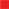), medaka (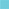), stickleback (*Gasterosteus aculeatus*; 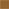), fugu (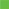), tetraodon (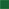) and zebrafish (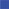). Lattice points containing no sequences after BLSOM calculation are indicated in white blank. 89, 95 and 97% of the sequences are located at the lattice points containing sequences from a single species (designated as pure lattice points) on DegDi, DegTri and DegTetra (see also Supplementary Fig. S1Ai and Bi). The separation of two closely related species, fugu and tetraodon, is rather poor especially on DegDi; therefore, black lattice points are evident for their territories (green for fugu and dark green for tetraodon). When these species belonging to Tetraodontidae are grouped into one category, 92, 97 and 98% of all fish sequences are located in the pure lattice points on DegDi, DegTri and DegTetra. BLSOM for 50-kb sequences also gave a clear species-specific separation (data not shown). (Aii) Examples of trinucleotides over-represented and evidently under-represented in the coelacanth genome. The occurrence of the trinucleotide for each lattice point has been calculated and normalized with the occurrence expected from the mononucleotide composition for each lattice point.^8^ This observed/expected ratio is indicated in colour presented at the bottom of the panel. (Bi) DegTri for 11 vertebrate genomes. Lattice points containing sequences from multiple species are indicated in black and those containing sequences from a single species are coloured. Fishes are marked as described in Ai, and tetrapods are as follows: chicken (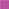), human (*Homo sapiens*; 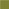), mouse (*Mus musculus*; 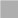), lizard (*Anolis carolinensis*; 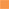) and *X. tropicalis* (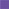). About 89, 97 and 98% of the sequences are located at the lattice points containing sequences from a single species (pure lattice points) on DegDi, DegTri and DegTetra, respectively (see also Fig. 2Ai and Supplementary Fig. S1Ci). When fugu and tetraodon are grouped into one category, 90, 98 and 99% of all fish sequences are located in the pure lattice points. (Bii) Lattice points containing sequences derived only from fish genomes are marked with the colours listed in Ai. (Biii) Examples of trinucleotides whose occurrence differs between coelacanth and other fishes. Lattice points are marked as described in Aii.

To compare the coelacanth's genome signature with that of other fishes and to study molecular evolutionary mechanisms for the establishment of their signatures, we constructed BLSOMs with di-, tri- and tetranucleotide compositions in all 100-kb sequence fragments from six fish genomes, including the African coelacanth genome. The fishes other than coelacanth were chosen, since a major portion of their genomes was sequenced and registered in the International DNA Sequence Databanks (DDBJ/EMBL/GenBank) as complete or draft genome sequences. In the Databanks, only one strand of complementary sequences is registered, and the strand is chosen rather arbitrarily in the registration of fragment sequences (e.g. draft genome sequences). Our previous BLSOM analysis of a wide range of species^7^ revealed that sequences from a single genome often gave a mirror-symmetrical split on BLSOM in the vertical direction, e.g. according to the replicational direction of genomic fragments.^13^ When investigating the general characteristics of genome sequences such as genome signatures, differences between two complementary strands are not important. Furthermore, the obtained map should not be affected by the choice of strands registered to the Databanks. Therefore, we constructed a BLSOM, for which the frequencies of a pair of complementary oligonucleotides in each fragment were summed up (e.g. AAC and GTT; abbreviated as AAC+GTT).^8^ The BLSOM for this degenerate set of a pair of complementary di-, tri- and tetranucleotides is abbreviated as DegDi, DegTri and DegTetra. The DegTri is listed in Fig. 1A; other two are listed in Supplementary Fig. S1A and B. Lattice points containing sequences from a single species are coloured to indicate the species, whereas those containing sequences from multiple species are indicated in black. With no information concerning species during BLSOM calculation, species-specific clustering (self-organization) of the fragment sequences is clear; i.e. sequences from individual species have formed their own territories. The number of black lattice points on DegTri and DegTetra is smaller than on DegDi, showing their higher clustering power; for details of their differences (Fig. 1A). One of the basal characteristics of BLSOM separation for genomic sequence has been attributed previously to the G+C%.^7,9,14^ Sequences with higher or lower G+C% are located on the left or right side of the map (Supplementary Figs S1Aii and S4Aii), showing that the G+C% is reflected primarily in the horizontal direction.

BLSOM can visualize diagnostic oligonucleotides responsible for species-specific clustering (self-organization) and provides information concerning possible molecular evolutionary mechanisms for establishing the present genome signatures. For this visualization, we have previously developed a method to unveil diagnostic oligonucleotides in a way unaffected by a simple difference in G+C% of genomic sequences.^8^ After calculating the frequency of each oligonucleotide expected from the mononucleotide composition for each lattice point, the observed/expected ratio is indicated in red (over-represented) or blue (under-represented) (Fig. 1Aii and Supplementary Fig. S1Aiii and Bii).

We focus on the oligonucleotides whose occurrence in coelacanth (red in Fig. 1Ai) differs from that in other fishes, i.e. the oligonucleotides, for which the transition between red and blue coincides with the border between the coelacanth's and the other fishes' territories in Fig. 1Aii. CCC+GGG is over-represented (red) only in the coelacanth territory; and CG-possessing trinucleotides are more under-represented in the coelacanth territory (dark blue) than in the other fishes' territories (pale blue). Importantly, all other CG-possessing trinucleotides (Supplementary Fig. S2A), as well as CG (Supplementary Fig. S1Aiii) and all CG-possessing tetranucleotides (Supplementary Fig. S2B), are more under-represented in the coelacanth territory (dark blue) than in the other fishes' territories (pale blue). This shows higher CG suppression in coelacanth than in other fishes; a numerical analysis of the CG suppression will be shown later.

### 3.2. BLSOM for a broad phylogenetic range of vertebrate genomes

Oligonucleotide BLSOM for fish genomes shows that a notable characteristic of coelacanth distinct from other fishes is the highest CG suppression. Importantly, this finding can be obtained with no prior knowledge, showing a prominent ability of this unsupervised data-mining method in knowledge discovery. Once this type of information is obtained, we can focus on detailed research of the respective genome characteristics. A major mechanism for the evident CG suppression in the mammalian genomes has been connected with methylation at C in CG dinucleotide, which is followed by spontaneous deamination. As a result, CG steadily mutates to TG, resulting in CG suppression and in CA and TG over-representation. In the case of fish genomes, by analysing a wide range of animal genomes from various aspects, Simmen^15^ has found that their CG suppression is also accountable in terms of 5-methylcytosine mutation; in the study, the author has proposed the following four criteria which support the 5-methylcytosine mutation. (i) Genome-level CG suppression. (ii) Genome-level TG and CA excess. (iii) Positive correlation between the local CG relative abundance (i.e. the observed/expected ratio of CG) and the local G+C% level. This is because regions of high G+C content possess a higher DNA melting temperature than low G+C content regions, and therefore, these are less susceptible to strand separation, resulting in a lower rate of deamination and a lesser degree of CG suppression. (iv) Negative correlation between local relative abundance of CG and TG (CA). We have found that all four criteria are applicable to coelacanth (Supplementary Fig. S3), showing that a major mechanism for the CG suppression in coelacanth is related to the 5-methylcytosine mutation, as previously reported for other fishes.^15^

**Figure 2.**
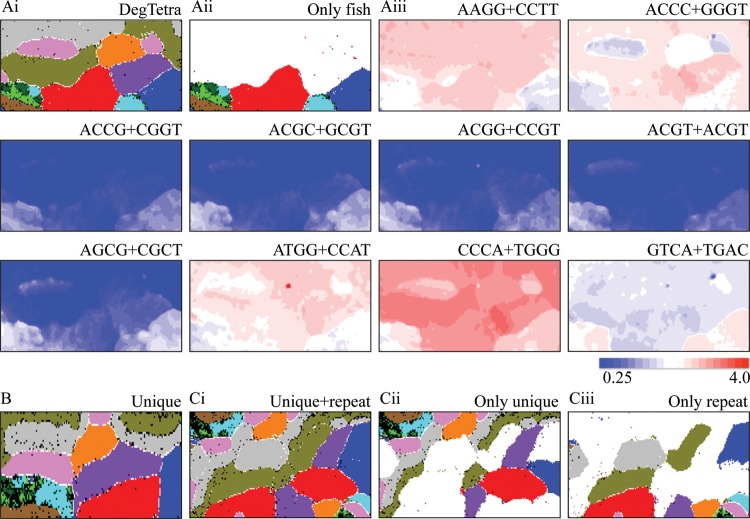
DegTetra for 100-kb sequences derived from 11 vertebrate genomes. (Ai and ii) Lattice points are marked as described in Fig. 1Bi and ii. (Aiii) Examples of tetranucleotides whose occurrence differs between coelacanth and other fishes. Lattice points are marked as described in Fig. 1Aii. (B) DegTri for 100-kb unique sequences from 11 vertebrate genomes. DegTri for repeat sequences also has shown the species-specific separation (data not shown). (Ci) DegTri constructed for unique plus repeat sequences. (Cii) Lattice points containing only unique sequences are marked. (Ciii) Lattice points containing only repeat sequences are marked.

Methylated C is a well-characterized epigenetic marker, and therefore, the highest CG suppression in coelacanth among fishes should be of particular interest in terms of evolutionary changes in epigenetic systems. We next included five additional vertebrate genomes covering a broad phylogenetic range of tetrapods, for which complete or draft genome sequences were available. On DegTri and DegTetra (Figs 1B and 2A), as well as DegDi (Supplementary Fig. S1C), for 100-kb sequences from the 11 vertebrate genomes, species-specific clustering is apparent. In Figs 1Bii and 2Aii, we mark the lattice points corresponding only to fish territories, which are located primarily in the lower part of the map.

The genomes of warm-blooded vertebrates are known to be composed of a long-range segmental G+C% distribution designated as ‘isochore’, which has been connected with chromosomal bands.^16–20^ A single genome, especially of a warm-blooded vertebrate, has multiple territories on DegTri [e.g. four chicken (*Gallus gallus*) territories marked in pink in Fig. 1Bi]. As previously mentioned, sequences with high or low G+C% are located on the left or right side of the map, and therefore, the split into several sub-territories in the horizontal direction should most likely relate to isochore structures.

On DegTri and DegTetra, the suppression of CG-possessing oligonucleotides in coelacanth and tetrapods and the high occurrence of CC+GG-possessing oligonucleotides in coelacanth and most tetrapods except chicken are clear (Figs 1Biii and 2Aiii); other examples of diagnostic oligonucleotides for phylotype-dependent clustering are listed in Supplementary Fig. S4. It should also be noted that the oligonucleotides diagnostic for one species are primarily common among the sub-territories of the species, e.g. the characteristic over-representation of CCC+GGG in the tetrapods other than chicken is observed regardless of intra-genomic G+C% differences such as isochores of chicken (Fig. 1Biii). In addition to the characteristic occurrence of CG- and CC+GG-possessing tetranucleotides, phylotype-dependent characteristics that cannot be explained by the characteristics of the dinucleotide composition are observed on DegTetra, e.g. under-representation of GTCA+TGAC in coelacanth and tetrapods (Fig. 2Aiii). Such oligonucleotides' biological significance will be discussed later.

### 3.3. Distinction between unique and repeat sequences

Higher eukaryote genomes are composed of unique and repeat sequences with different biological significances. Since repeat sequences usually have peculiar oligonucleotide composition, the composition in repeat sequences might be a major causal factor for genome signatures detected by the present oligonucleotide BLSOMs. This possibility is tested as follows. In the UCSC Genome Browser, repeat sequences identified by RepeatMasker and Tandem Repeats Finder (with a period of ≤12) are specified in lower-case letters to distinguish from unique sequences specified in upper-case letters. We thus separately constructed oligonucleotide BLSOMs for repeat and unique sequences of the 11 vertebrates; we first concatenated repeat and unique sequences separately and divided these artificially concatenated sequences into 100-kb fragments, followed by counting oligonucleotide composition. DegTri for unique sequences shows the clear species-specific separation (Fig. 2B), proving that their genome signatures are not the peculiar characteristics of oligonucleotide compositions in repeat sequences; the same finding was obtained on DegDi and DegTetra (data not shown). In Fig. 2Ci, unique plus repeat sequences have been analysed on one DegTri; using the same map, unique and repeat sequences are separately marked (Fig. 2Cii and iii). Clear separation is observed among species and between unique and repeat sequences, showing differential oligonucleotide compositions among these different categories.

### 3.4. CG suppression in a wide phylogenetic range of vertebrates

**Figure 3.**
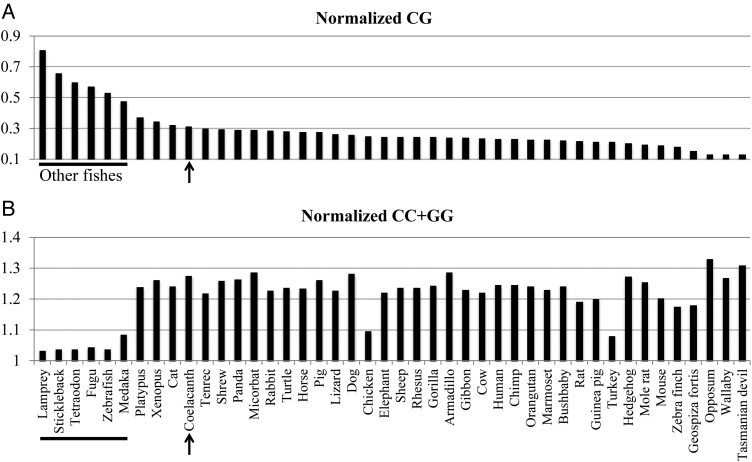
Normalized CG and CC+GG levels for 44 vertebrate genomes: Armadillo (*Dasypus novemcinctus*), bushbaby (*Otolemur garnettii*), cat (*F. catus*), chicken (*G. gallus*), chimp (*Pan troglodytes*), coelacanth (*L. chalumnae*), cow (*Bos taurus*), dog (*Canis lupus*), elephant (*Loxodonta africana*), fugu (*Fugu rubripes*), Geospiza fortis (*Geospiza fortis*), Gibbon (*Nomascus leucogenys*), Gorilla (*Gorilla gorilla*), Guinea pig (*Cavia porcellus*), Hedgehog (*Erinaceus europaeus*), horse (*Equus caballus*), human (*H. sapiens*), lamprey (*P. marinus*), lizard (*A. carolinensis*), marmoset (*Callithrix jacchus*), medaka (*O. latipes*), micorbat (*Myotis lucifugus*), mole rat (*Heterocephalus glaber*), mouse (*M. musculus*), opposum (*M. domestica*), orangutan (*Pongo pygmaeus*), panda (*Ailuropoda melanoleuca*), pig (*Sus scrofa*), platypus (*O. anatinus*), rabbit (*Oryctolagus cuniculus*), rat (*Rattus norvegicus*), rhesus (*Macaca mulatta*), sheep (*Ovis aries*), shrew (*Sorex araneus*), stickleback (*G. aculeatus*), tasmanian devil (*S. harrisii*), tenrec (*E. telfairii*), tetraodon (*Tetraodon nigroviridis*), Turkey (*M. gallopavo*), turtle (*Chrysemys picta*), wallaby (*M. eugenii*), Xenopus (*X. tropicalis*), Zebra finch (*Taeniopygia guttata*), and Zebrafish (*D. rerio*). (A and B) The occurrence level of CG and of CC+GG normalized with the level expected from the mononucleotide composition. The species are arranged in descending order of the normalized CG level. The data for coelacanth is arrowed and those for other fishes are indicated by a horizontal bar.

A difference in CG suppression observed among different lineages of vertebrates may relate to the differential features in epigenetic systems.^21,22^ Considering the fact that biologically important genome characteristics tend to be maintained stably during evolution, we analysed the CG suppression of many more vertebrates than those analysed with BLSOMs, because CG suppression can be analysed even if their contiguous sequences are rather short (e.g. shorter than 100 kb). Specifically, we analysed CG suppression for 44 vertebrate genomes covering a broad phylogenetic range. The CG/GC ratio (often called the CpG/GpC ratio) has long been used to examine CG suppression for individual species, because this ratio is not affected by the genome G+C% difference. Strictly speaking, however, this ratio should be affected by the possible species-specific GC dinucleotide occurrence. Therefore, the observed/expected ratio of CG (i.e. normalization with the occurrence level expected from the mononucleotide composition) was analysed (Fig. 3A); the species are arranged in descending order of the normalized CG level. Supporting the BLSOM results, the normalized CG level in coelacanth (0.31, arrowed) is clearly lower than in other fishes (indicated by a horizontal bar) and rather equivalent to that of a wide range of tetrapods, e.g. platypus (*Ornithorhynchus anatinus*; 0.38), *X. tropicalis* (*Xenopus tropicalis*; 0.35), cat (*Felis catus*; 0.33) and tenrec (*Echinops telfairii*; 0.30). It should also be mentioned that Marsupialia, such as tasmanian devil (*Sarcophilus harrisii*), wallaby (*Macropus eugenii*) and opossum (*Monodelphis domestica*), have a very low-CG occurrence (<0.14). Since the CG suppression has been evolutionarily conserved well within individual phylogenetic lineages, the CG suppression is thought to relate to fundamental biological functions. Similar results are found for the aforementioned CG/GC ratio (Supplementary Fig. S5A). Supplementary Fig. S5A also shows that the evident CG suppression of coelacanth among fishes is observed in both unique and repeat sequences. Supplementary Fig. S5B and C shows that CA/AC and TG/GT ratios of coelacanth are higher than those of other fishes and equivalent to most of tetrapods, as expected from the mechanism responsible for the CG suppression; CA and TG over-representation.

**Figure 4.**
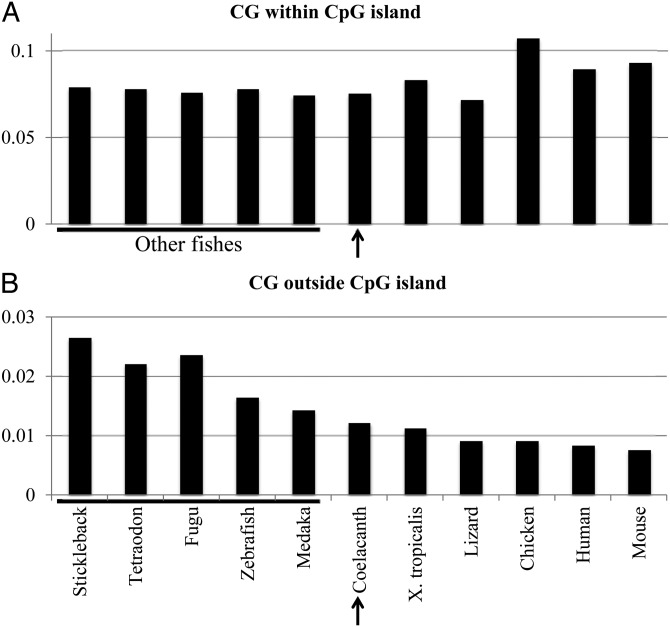
CG occurrence within and outside CpG islands for 11 vertebrate genomes analysed in Fig. 2.

CpG islands, which belong to the unique sequence category, have been connected with transcriptional regulatory mechanisms. Figure 4 analysed the CG level in the sequences within and outside CpG islands, which were identified by ‘newcpgreport’ (http://emboss.sourceforge.net/apps/cvs/emboss/apps/newcpgreport.htm). The CG level outside CpG islands varies significantly among fishes and the coelacanth has the lowest CG level, as reflecting its global genome characteristics. In contrast, the CG level within CpG islands is rather similar among fishes including coelacanth. This level varies significantly among tetrapods; the level of lizard is similar to that of coelacanth, but those of chicken, mouse and human are clearly higher than that of coelacanth. This difference observed even between closely related species may reflect the difference in actual detailed molecular mechanisms in the transcriptional regulation. The higher CG suppression outside CpG islands (Fig. 4B) is consistent with the hypothesis that hypermutation of methylated C is the main cause of higher CG suppression in coelacanth, because methylation of C generally occurs outside CpG islands.

### 3.5. DNA methylase genes

The CG occurrence in lamprey (*Petromyzon marinus*) is the highest (0.78) and rather similar to a level of the very slightly methylated genome of drosophila (*Drosophila melanogaster*),^23,24^ but significantly differs from medaka (*Oryzias latipes*; 0.46) (Fig. 3A). This wide range of CG suppression among fishes indicates that various changes in DNA-methylation and -demethylation systems have occurred during the fish lineage evolution. Taking this into account, we next consider the possible molecular mechanisms responsible for the highest CG suppression of coelacanth among fishes. Since CG suppression in vertebrate genomes is believed to relate to methylation at CG dinucleotide, we first compared *de novo* DNA methylases found in the coelacanth genome with those of other fishes and tetrapods; *de novo* DNA methylases are responsible for the new addition of a methyl group to DNA. As opposed to the two catalytically active *de novo* DNA methylase genes (*dnmt3a* and *dnmt3b*) of mammals, zebrafish (*Danio rerio*) is known to have six *de novo* DNA methylase genes and lacks *dnmt3l* which is catalytically inactive but required for the establishment of genomic imprinting.^25,26^ It is also known that the expression pattern of zebrafish *dnmt6* and *dnmt8*, which more closely resemble mammalian *dnmt3a* in amino acid sequence, more closely resembles that of mammalian *dnmt3a* than *dnmt3b*, but the expression pattern of the other four zebrafish genes (*dnmt3*, *dnmt4*, *dnmt5* and *dnmt7*) resembles the pattern of mammalian *dnmt3b*, i.e. *dnmt6* and *dnmt8* have been shown to more ubiquitously express in developing and adult tissues than the other four zebrafish genes.^27^

**Figure 5.**
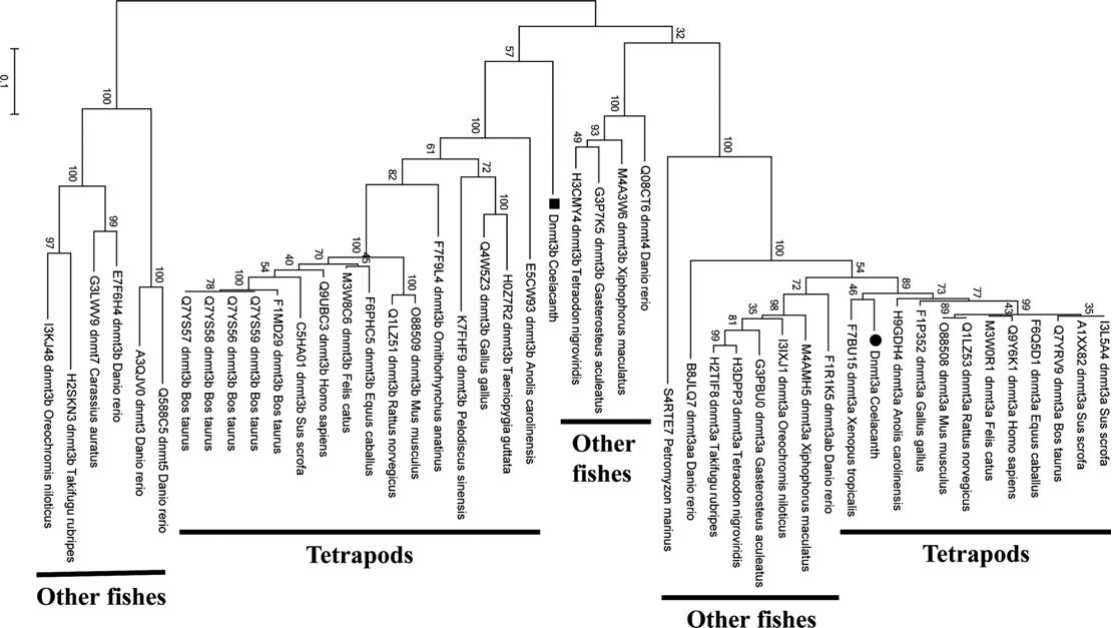
Phylogenetic tree of Dnmt3 constructed with maximum likelihood. Nine hundred and fifty amino acid sites are used for the tree inference and the tree is unrooted. A bootstrap value is presented above each branch. The scale is proportional to the number of substitutions per amino acid.

**Figure 6.**
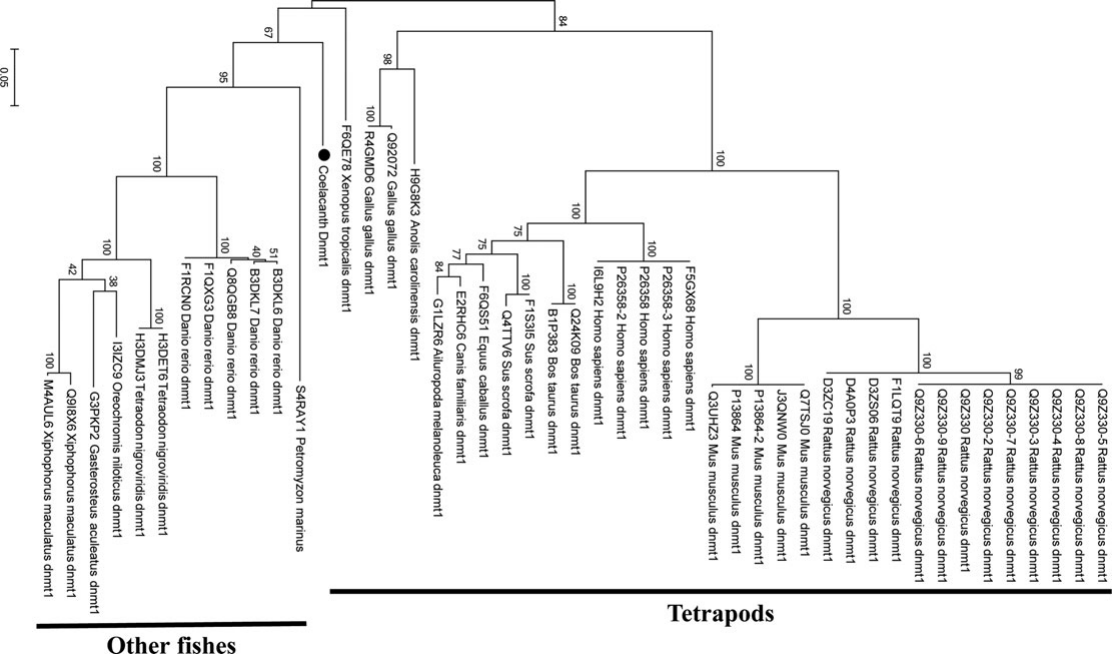
Phylogenetic tree of Dnmt1 constructed with maximum likelihood. One thousand five hundred and twenty amino acid sites are used for the tree inference and the tree is unrooted. A similar tree was obtained with neighbour-joining and minimum evolution (data not shown).

In the coelacanth genome, we can find two *dnmt3* genes (*dnmt3a* and *dnmt3b*) as observed for mammals but no *dnmt3l* as observed for zebrafish. Using multiple sequence alignment (MUSCLE) in Mega6,^28^ phylogenetic analyses of coelacanth Dnmt3a and Dnmt3b were conducted at a protein-sequence level (Fig. 5 and Supplementary Fig. S6). All three programmes (maximum likelihood, minimum evolution and neighbour-joining) show that the coelacanth Dnmt3a is clustered with tetrapods' Dnmt3a, but not with other fishes' Dnmt3a. In the case of Dnmt3b, maximum likelihood shows that the coelacanth Dnmt3b is clustered with tetrapods' Dnmt3b as observed for Dnmt3a (Fig. 5), but minimum evolution and neighbour-joining show it to be outside of tetrapods' and other fishes' clusters (Supplementary Fig. S6). In the case of Dnmt1, which preferentially methylates hemimethylated DNA for maintaining the methylation pattern in the newly synthesized strand, all three programmes show the coelacanth Dnmt1 to be outside of tetrapods' and other fishes' clusters (Fig. 6).

The difference found among these three methylases, which have differential biological functions, may indicate the differential level of contribution to the CG suppression during evolution; the ubiquitously expressed *de novo* methylase Dnmt3a may have contributed most significantly to the evident CG suppression in coelacanth. For the adequate understanding of CG suppression, however, additional molecular mechanisms such as RNA-directed DNA methylation^29^ and DNA-demethylation systems have to be considered. Since the CG-suppression level has stably been conserved phylogenetically (Fig. 3 and Supplementary Fig. S5), analyses of this level from various aspects may provide novel knowledge about fundamental biological mechanisms related to epigenetics.

### 3.6. Oligonucleotides unrelated to CG

If we know in advance that a fundamental characteristic of the coelacanth genome distinct from other fishes is related to CG suppression, a simple analysis of dinucleotide composition should be enough to prove this characteristic. However, when we have not such background knowledge, an unsupervised data-mining method such as the present BLSOM becomes valuable because it can unveil a wide range of characteristics hidden in the genome sequence. Actually, the occurrence of various CG-non-possessing oligonucleotides in coelacanth was found distinct from that in other fishes, but equivalent to that in almost all tetrapods. A clear example is the over-representation of (CC+GG)-possessing tri-and tetranucleotides on DegTri and DegTetra (Figs 1Biii and 2Aiii). Figure 3B presents the observed/expected ratio of CC+GG in 44 vertebrate genomes, and Supplementary Fig. S7A and B presents the results for CCC+GGG and CCCC+GGGG. As observed on BLSOMs (Fig. 1Aii and Biii, and Supplementary Fig. S1Aiii, Bii and Ciii), their occurrences in coelacanth are clearly higher than in other fishes. The same is true for most tetrapods, including *Marsupialia* but excluding chicken and turkey (*Meleagris gallopavo*). It should also be noted that the occurrence of CCCC+GGGG in coelacanth is the highest among the vertebrates analysed (Supplementary Fig. S7B).

## 4. Discussion

The genome characteristics maintained stably in a certain phylogenetic lineage should reflect its fundamental biological significance. An apparent causative factor for the genome signature is the context-dependent DNA mutation and repair mechanisms. It should also be mentioned that the oligonucleotides longer than tetranucleotides often represent motif sequences responsible for sequence-specific protein binding (e.g. transcription factor binding). The occurrence of such motif oligonucleotides should differ from the level expected from the mononucleotide composition in the respective genome and may differ among genomic portions within one genome. We have recently found that DegPenta- and DegHexa-BLSOMs for the human genome can effectively detect characteristic enrichment of many transcription factor-binding motifs in pericentric heterochromatin regions.^30^ The present study has found evident CG suppression and CC+GG over-representation in coelacanth and tetrapods, by using DegDi, DegTri and DegTetra. If the BLSOM analysis extends to oligonucleotides longer than tetranucleotides, we may assign *in silico* the oligonucleotides with important biological functions such as transcription factor-binding in the genome of coelacanth and other species, for which experimental studies other than DNA sequencing have been poorly done.

BLSOM can analyse big sequence data such as millions of sequences simultaneously^31^ and, therefore, can analyse genome sequences from a wide range of species and visualize characteristics of their oligonucleotide composition on a single map. This can support an efficient knowledge discovery from the big data with no prior knowledge. Pointing out the oligonucleotides whose occurrence is peculiar commonly (and thus stably) in a certain lineage should provide information concerning biological roles of these oligonucleotides in the lineage. In conclusion, BLSOM is a powerful and timely tool useful for genome studies in the era of big sequence data obtained from high-throughput DNA sequencers.

*Conflict of Interest statement.* None declared.

## Supplementary Data

Supplementary Data are available at www.dnaresearch.oxfordjournals.org.

## Funding

This work was supported by Grant-in-Aid for Scientific Research (C) and for Young Scientists (B) from the Ministry of Education, Culture, Sports, Science and Technology of Japan and by the Grant-in-Aid for JSPS Fellows (JSPS KAKENHI Number 24-9979). The computation was done in part with the Earth Simulator of Japan Agency for Marine-Earth Science and Technology.

## Supplementary Material

Supplementary Data
