# Supplementary material for: Evolutionary Changes in Vertebrate Genome Signatures with Special Focus on Coelacanth
Source: DNA Res. 2014 May 6;21(5):459–67. doi: 10.1093/dnares/dsu012 (PMC4195492; doi:10.1093/dnares/dsu012)
Supplement: Supplementary Data [file supp_21_5_459__index.html]

Evolutionary Changes in Vertebrate Genome Signatures with Special Focus on Coelacanth — Evolutionary Changes in Vertebrate Genome Signatures with Special Focus on Coelacanth — Supplementary Data 

# Evolutionary Changes in Vertebrate Genome Signatures with Special Focus on Coelacanth

## Supplementary Data

Supplementary Data

**Files in this Data Supplement:**

- Supplementary Data - Docx file
- Supplementary Figures - ppt file
