# Supplementary material for: Evolutionary Changes in Vertebrate Genome Signatures with Special Focus on Coelacanth
Source: DNA Res. 2014 May 6;21(5):459–67. doi: 10.1093/dnares/dsu012 (PMC4195492; doi:10.1093/dnares/dsu012)
Supplement: Supplementary Data [file supp_dsu012_dsu012supp.docx]

**Supplementary Data**

**Figure S1.** BLSOM for 100-kb sequences. (Ai) DegDi for six fish genomes. Lattice points are colored as described in Fig. 1Ai. (Aii) G+C%. The G+C% obtained for each lattice point on DegDi is divided into 21 groups containing an equal number of lattice points^14^, and the highest and lowest groups are shown in dark wine red and dark green, respectively. Sequences with higher or lower G+C% (wine red or green) are located on the left or right side of the map, showing that the G+C% is reflected primarily in the horizontal direction; the same is true for DegTri and DegTetra (data not shown). (Aiii) Examples of dinucleotides overrepresented and evidently underrepresented in the coelacanth genome. (Bi) DegTetra for six fish genomes. (Bii) Examples of diagnostic tetranucleotides for phylotype-dependent clustering. (C) DegDi for 100-kb sequences from 11 vertebrate genomes.

**Figure S2.** BLSOM for six fish genomes. Examples of tri- and tetranucleotides (A and B) diagnostic for the phylotype-specific clustering found on DegTri (Fig. 1Ai) and DegTetra (Fig. S1Bi). Other CG possessing tetranucleotides are evidently underrepresented in the coelacanth genome (data not shown).

**Figure S3.** Normalized CG and CA+TG levels against G+C% in the coelacanth genome. The occurrence level of CG and of CA+TG normalized with the level expected from the mononucleotide composition is plotted according to Simmen (2008).^15^ Each point represents the data from a single 50-kb genomic segment; black and blue dots represent the normalized CG and CA+TG, respectively. Almost all dots representing CG and CA+TG are located below and above the line representing 1.0, respectively, supporting the criterion 1 and 2 listed in the text. Positive (r = 0.75) and negative (r = -0.42) correlations are found between CG and G+C% and between CA+TG and G+C%, respectively, and therefore, a negative correlation (r = -0.70) is found between CG and CA+TG. These findings support the criterion 3 and 4.

**Figure S4.** BLSOM for 11 vertebrate genomes. (Ai) DegDi 11. Lattice points are colored as described in Fig. 1Bi. (Aii) G+C% of each lattice point is shown as described in Fig. S1Aii. Examples of tri- and tetranucleotides (**B** and **C**) diagnostic for the phylotype-specific clustering found on DegTri (Fig. 1Bi) and DegTetra (Fig. 2Ai).

**Figure S5.** CG/GC, CA/AC and TG/GT ratios for 44 vertebrate genomes (A, B and C). Blue, red and green bars show the ratio for repeat, unique and a total genome, respectively. The species are arranged in descending order of the normalized CG level, as described in Fig. 3. The data for coelacanth is arrowed.

**Figure S6.** Phylogenetic tree of Dnmt3 constructed with **Neighbor-joining**. Bootstrap value is presented above each branch. The scale is proportional to the number of substitutions per amino acid. This tree is very similar to that constructed with **Minimum Evolution** (data not shown).

**Figure S7.** Normalized occurrence levels of CCC+GGG (A) and CCCC+GGGG (B) for 44 vertebrates. The species are arranged in descending order of the normalized CG level, as described in Fig. 3. The data for coelacanth is arrowed.
