# Supplementary material for: Evolutionary Changes in Vertebrate Genome Signatures with Special Focus on Coelacanth
Source: DNA Res. 2014 May 6;21(5):459–67. doi: 10.1093/dnares/dsu012 (PMC4195492; doi:10.1093/dnares/dsu012)
Supplement: Supplementary Data [file supp_dsu012_dsu012supp_figs.ppt]

## Slide 1
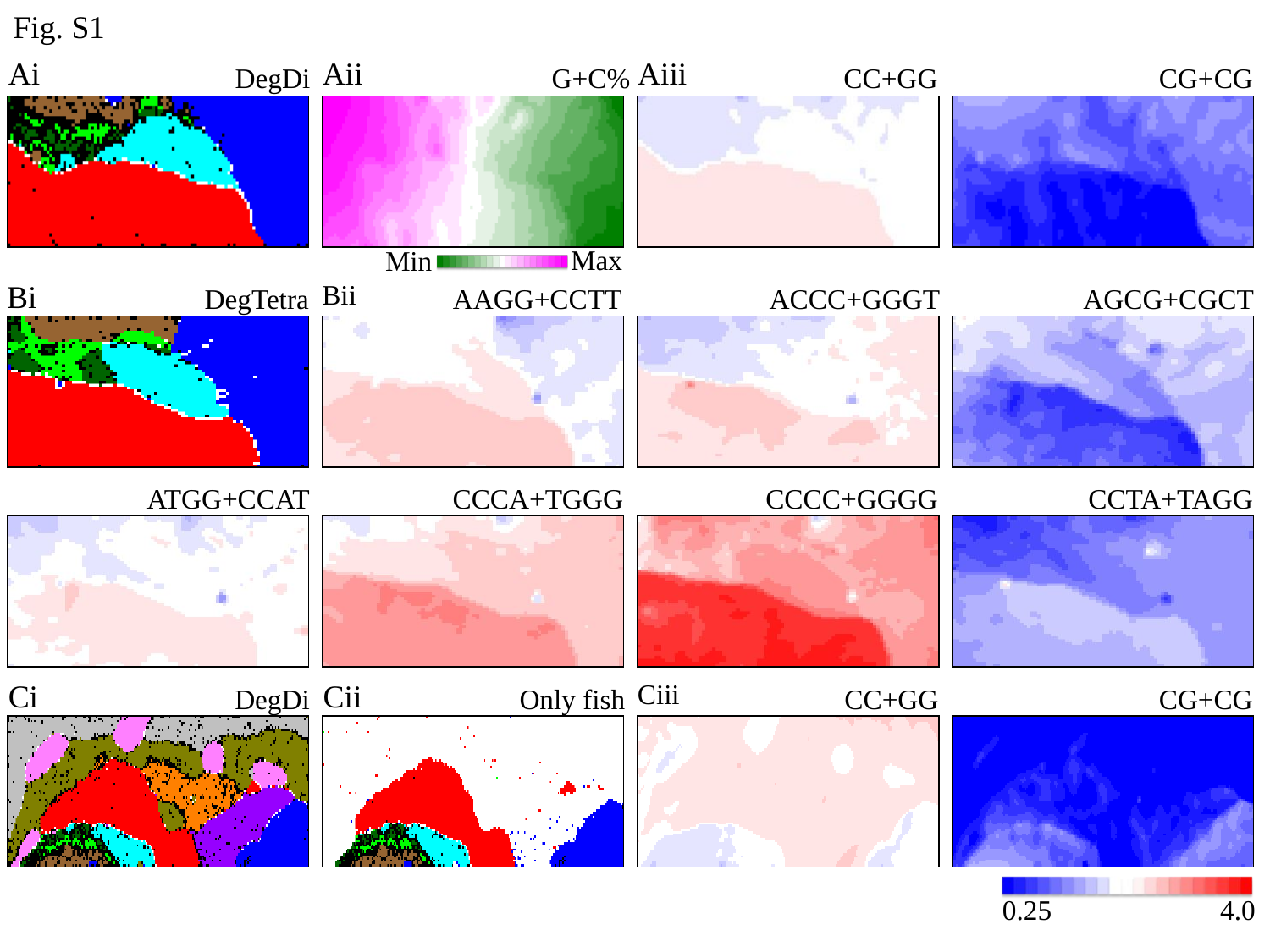

Fig. S1
Ai
Aii
Aiii
DegDi
　G+C%
CC+GG
CG+CG
Max
Min
Bi
Bii
DegTetra
AAGG+CCTT
ACCC+GGGT
AGCG+CGCT
ATGG+CCAT
CCCA+TGGG
CCCC+GGGG
CCTA+TAGG
Ci
Cii
Ciii
DegDi
Only fish
CC+GG
CG+CG
0.25
4.0

## Slide 2
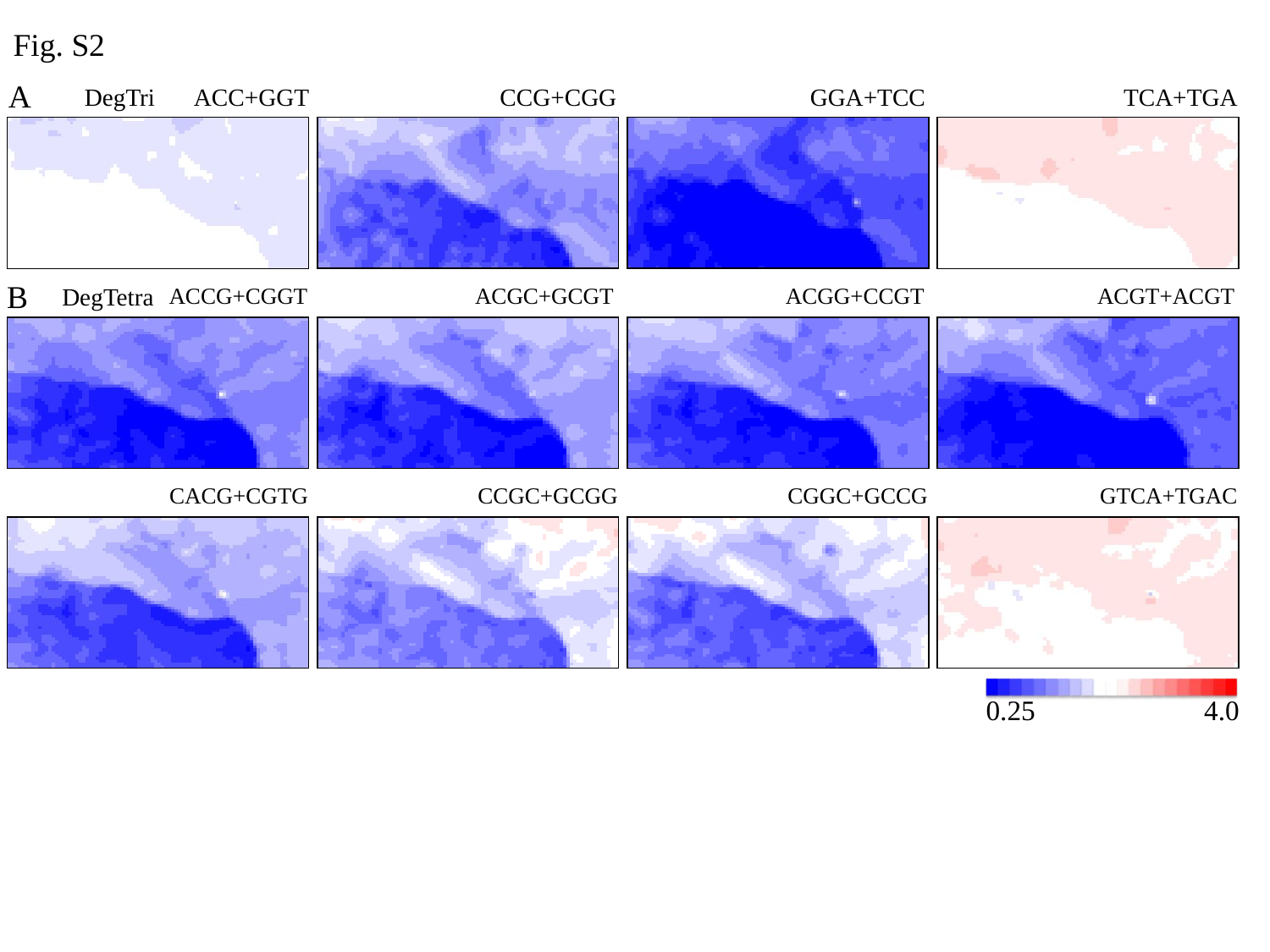

Fig. S2
A
DegTri
ACC+GGT
CCG+CGG
GGA+TCC
TCA+TGA
B
DegTetra
ACCG+CGGT
ACGC+GCGT
ACGG+CCGT
ACGT+ACGT
CACG+CGTG
CCGC+GCGG
CGGC+GCCG
GTCA+TGAC
0.25
4.0

## Slide 3
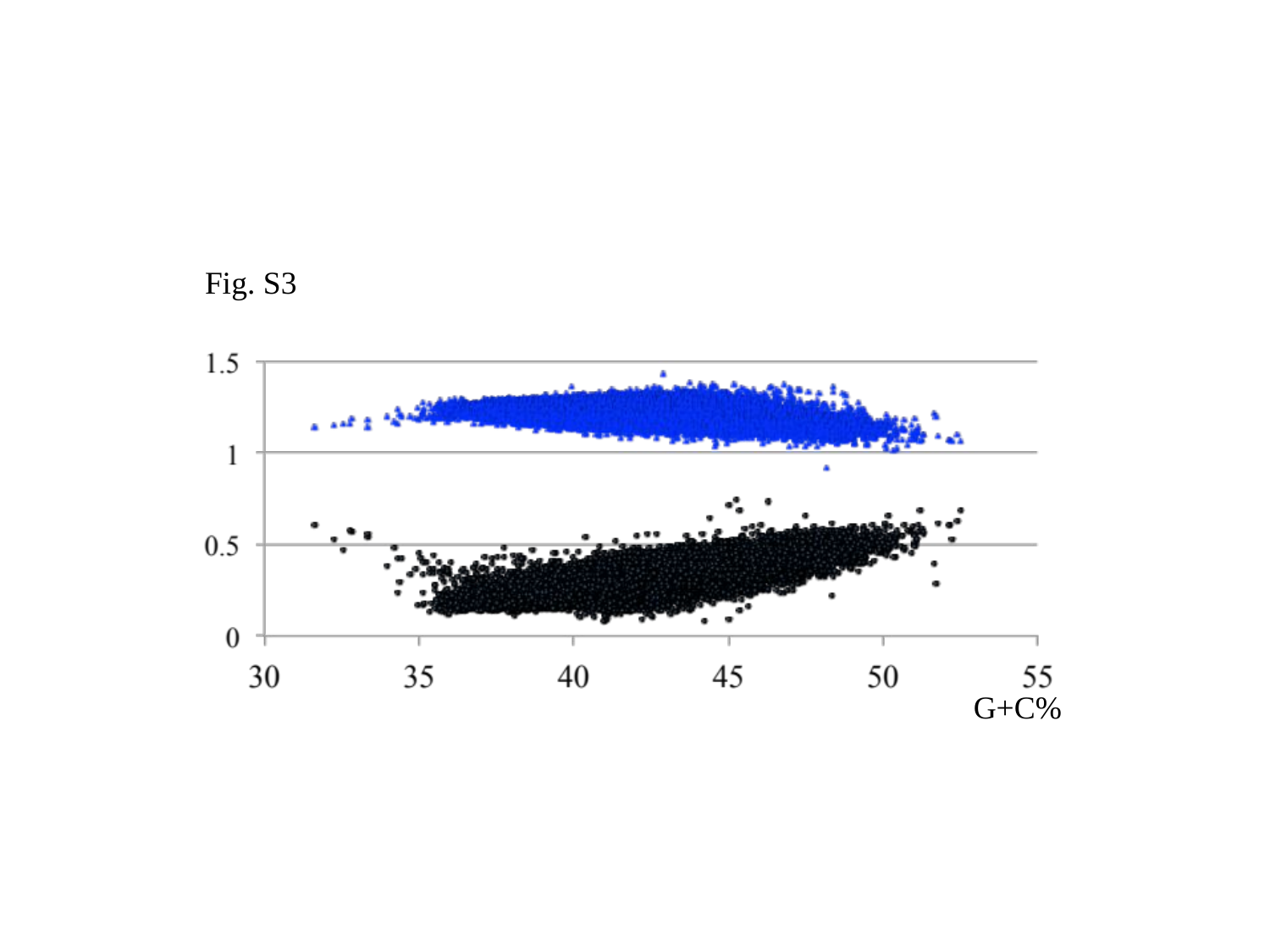

Fig. S3
G+C%

## Slide 4
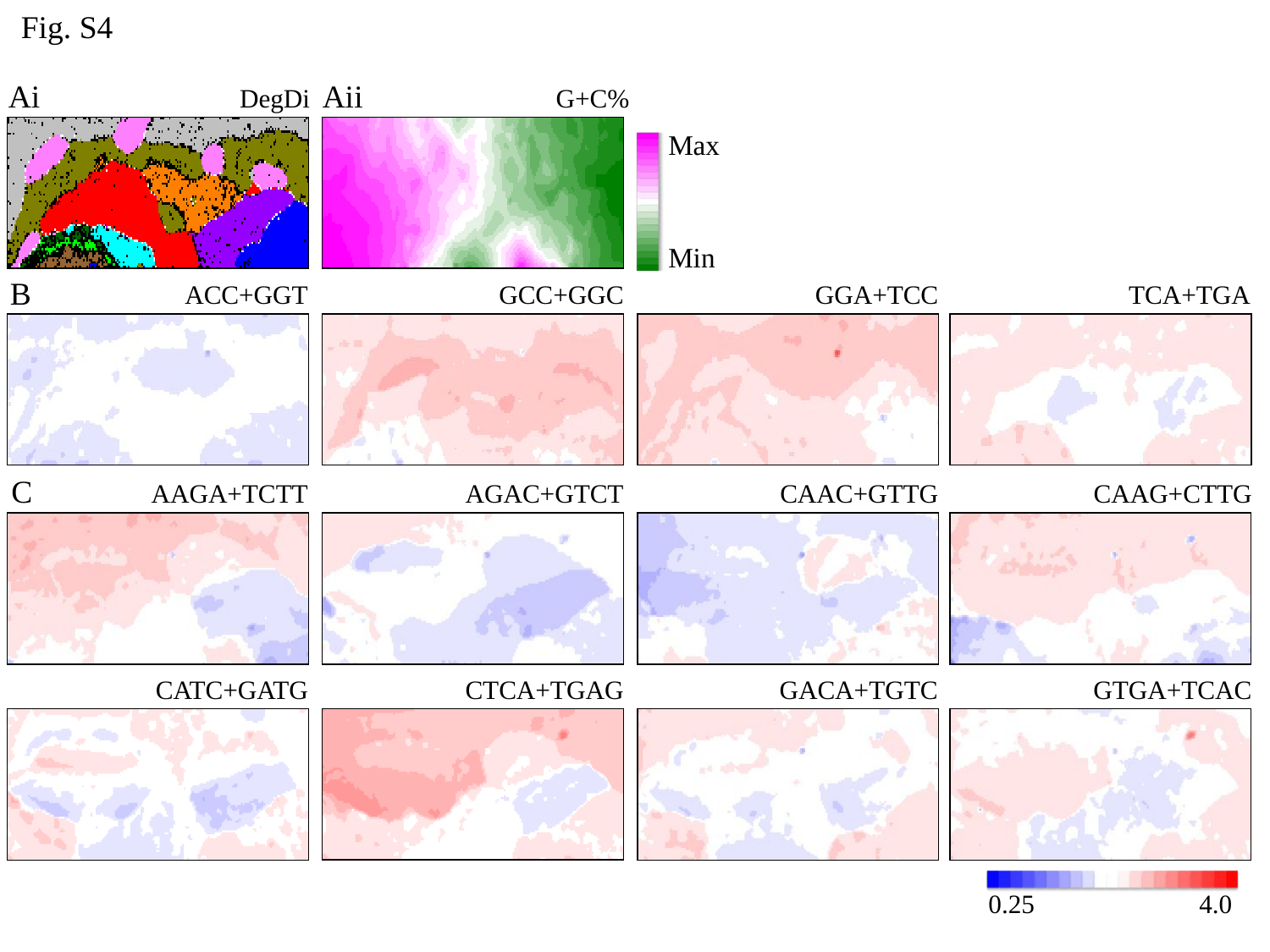

Fig. S4
Ai
Aii
DegDi
　G+C%
Max
Min
B
ACC+GGT
GCC+GGC
GGA+TCC
TCA+TGA
C
AAGA+TCTT
AGAC+GTCT
CAAC+GTTG
CAAG+CTTG
CATC+GATG
CTCA+TGAG
GACA+TGTC
GTGA+TCAC
0.25
4.0

## Slide 5
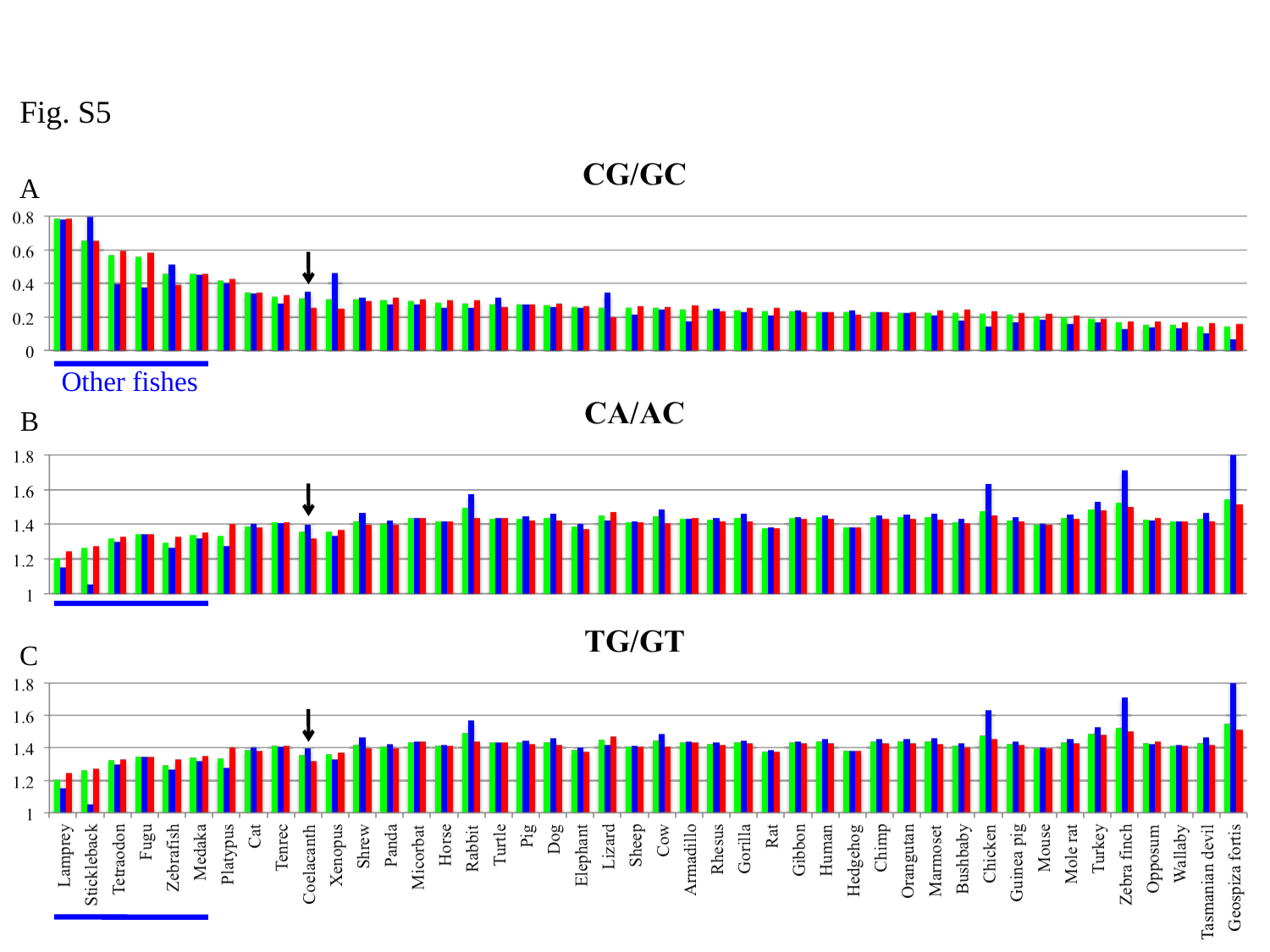

Fig. S5
A
Other fishes
B
C

## Slide 6
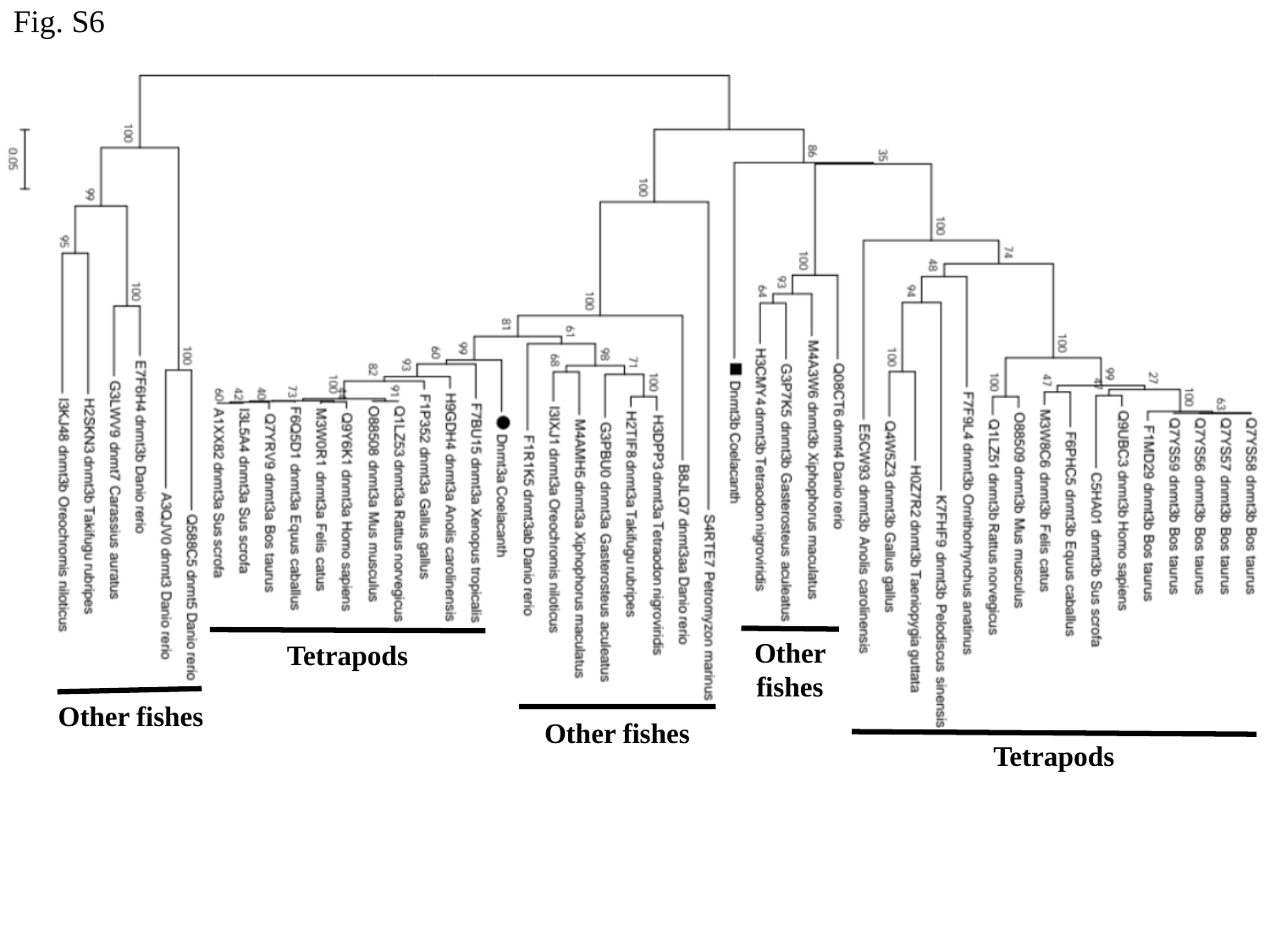

Fig. S6
Other
fishes
Tetrapods
Other fishes
Other fishes
Tetrapods

## Slide 7
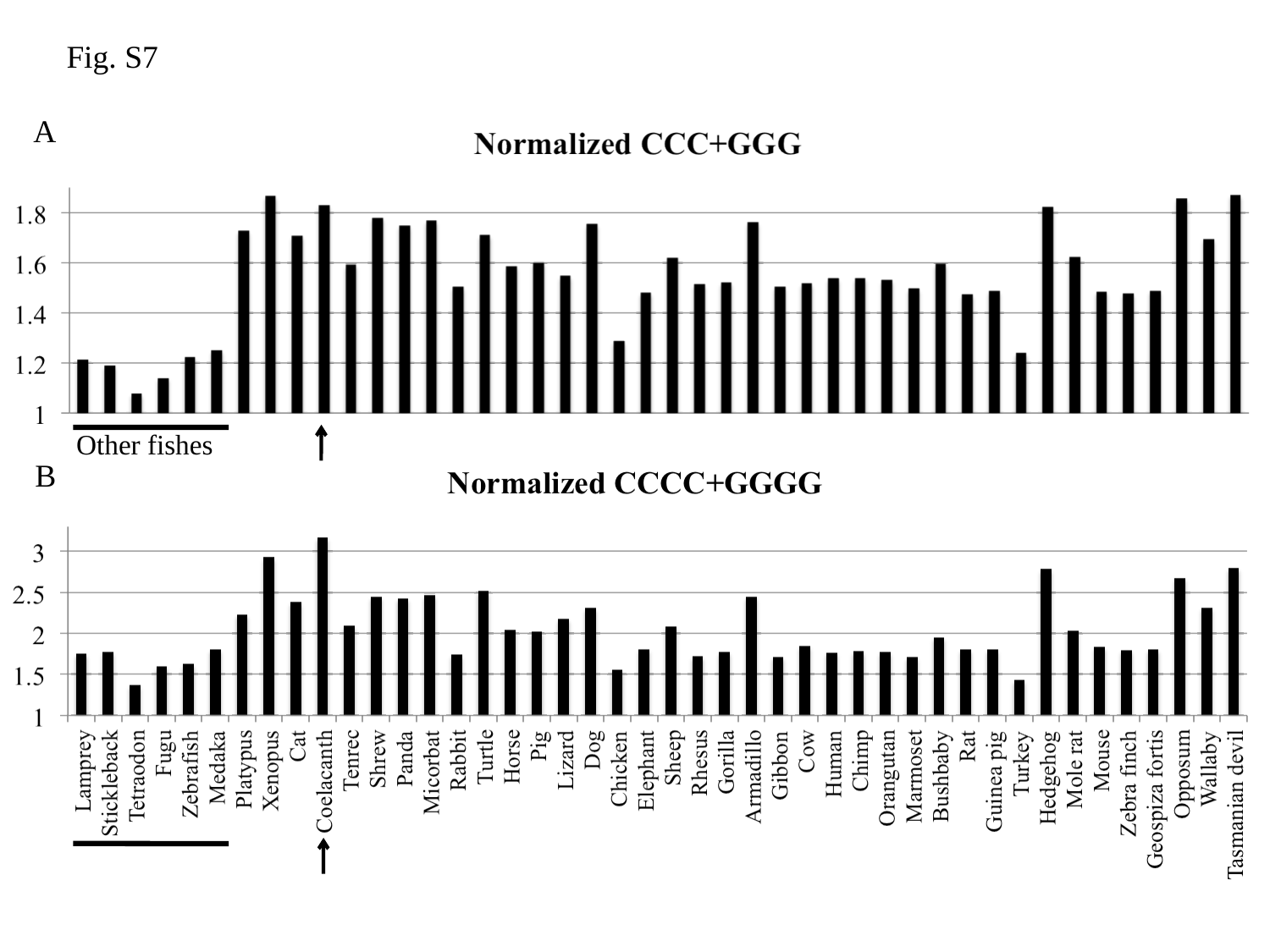

Fig. S7
A
Other fishes
B
